# Anti-Bacterial Adhesion Activity of Tropical Microalgae Extracts

**DOI:** 10.3390/molecules23092180

**Published:** 2018-08-29

**Authors:** Claudia Zea-Obando, Alina Tunin-Ley, Jean Turquet, Gérald Culioli, Jean-François Briand, Alexis Bazire, Karine Réhel, Fabienne Faÿ, Isabelle Linossier

**Affiliations:** 1 Institut Européen de la Mer, Université de Bretagne-Sud, EA 3884, LBCM, F-56100 Lorient, France; claudia.zea@univ-ubs.fr (C.Z.-O.); alexis.bazire@univ-ubs.fr (A.B.); karine.rehel@univ-ubs.fr (K.R.); isabelle.linossier@univ-ubs.fr (I.L.); 2 Laboratory c/o CYROL, NEXA, 97490 Sainte Clotilde, Reunion, France; Alina.tunin-ley@hydroreunion.re (A.T.-L.); jean.turquet@hydroreunion.re (J.T.); 3 MAPIEM, Biofouling et Substances Naturelles Marines, Université du Sud Toulon-Var, EA 4323, 83041 Toulon, France; culioli@univ-tln.fr (G.C.); briand@univ-tln.fr (J.-F.B.)

**Keywords:** tropical microalgae, antiadhesion activity, marine bacteria, dinoflagellates, *Symbiodinium*

## Abstract

The evolution of regulations concerning biocidal products aimed towards an increased protection of the environment (e.g., EU Regulation No 528/2012) requires the development of new non-toxic anti-fouling (AF) systems. As the marine environment is an important source of inspiration, such AF systems inhibiting the adhesion of organisms without any toxicity could be based on molecules of natural origin. In this context, the antibiofilm potential of tropical microalgal extracts was investigated. The tropics are particularly interesting in terms of solar energy and temperatures which provide a wide marine diversity and a high production of microalgae. Twenty microalgal strains isolated from the Indian Ocean were studied. Their extracts were characterized in terms of global chemical composition by high resolution magic angle spinning (HR-MAS) and nuclear magnetic resonance (NMR) spectroscopy, toxicity against marine bacteria (viability and growth) and anti-adhesion effect. The different observations made by confocal laser scanning microscopy (CLSM) showed a significant activity of three extracts from Dinoflagellate strains against the settlement of selected marine bacteria without any toxicity at a concentration of 50 μg/mL. The *Symbiodinium* sp. (P-78) extract inhibited the adhesion of *Bacillus* sp. 4J6 (Atlantic Ocean), *Shewanella* sp. MVV1 (Indian Ocean) and *Pseudoalteromonas lipolytica* TC8 (Mediterranean Ocean) at 60, 76 and 52%, respectively. These results underlined the potential of using microalgal extracts to repel fouling organisms.

## 1. Introduction

The recent regulation concerning the use of biocidal agents (EU Regulation No 528/2012 known as Biocidal Product Regulation (PBR)) in antifouling (AF) systems requires a higher level of environmental protection [1]. Hence, new AF compounds without toxic effects are needed. Among the proposed strategies, the use of AF natural products from terrestrial or marine resources is widely documented [2,3]. For example, Chen et al. have recently proposed the incorporation of butenolide, a furanone derivative isolated from a marine *Streptomyces*, in various AF paint formulations [4]. This natural compound has shown a strong antifouling activity against larval settlement of barnacles, bryozoans and polychaetes with low toxicity [5,6,7]. 

Indeed, marine organisms are an exceptional source of bioactive metabolites. The chemical diversity of the molecules they produce as well as the wide range of biological activities they show (e.g., antibiotics, toxins, antibiofilm agents, UV protective compounds, immune modulators) make them very attractive [8]. Among marine organisms, bacteria, fungi, microalgae, cyanobacteria and their symbiotic associations have increasingly become sources of such compounds [9]. More precisely, a large number of microalgal extracts have demonstrated antibacterial, antifungal, anti-algal and antiprotozoal activities [10,11,12]. Microalgae contain a high number of active compounds from diverse chemical families (e.g., fatty such as fatty acids derivatives, peptides, terpenoids, polysaccharides) which combat bacterial colonization [10,11,13]. For example, Desbois et al. [14] isolated an antibacterial polyunsaturated fatty acid from the marine diatom, *Phaeodactylum tricornutum*, which showed an activity against a range of both Gram-positive and Gram-negative bacteria. Hence, microalgal extracts are particularly interesting to inhibit the development of biofilm and biofouling, especially as they could be obtained in large amounts and optimized by means of biotechnological process [15].

Antifouling compounds act on target organisms through multiple pathways, among which surface modifiers and inhibitors of bacterial quorum sensing and biofilms formation stand out [16]. A recent review summarizes the activity of zosteric acid, a phenolic compound extracted from the marine sea grass *Zostera marina* [17]. Several mechanisms have been identified, among which, the prevention of the formation of biofilms by blocking the surface attachment sites of bacteria may be highlighted. Capsaicin isolated from the chili pepper, *Capsicum frutescens* L., is believed to act in a similar way [18].

The geographical origin of the studied microalgae is very varied. However, due to high solar irradiance and temperature, the tropics provide wide variety and industrial mass production capability of marine microalgae [19,20,21]. It has been shown that tropical microalgae produce a variety of lipids for biodiesels and nutraceutical [22,23]. Nevertheless, despite the abundant literature on the antibacterial activity of various organisms from Indian Ocean [24,25,26,27,28,29], no study reports the use of tropical microalgae as a source of anti-bioadhesion agents against fouling bacteria. 

This work studied the use of tropical microalgal extracts as anti-bioadhesion ingredients. Twenty methanolic extracts of tropical microalgae have been evaluated against eight marine bacteria isolated from surfaces immersed in Atlantic and Indian Oceans and the Mediterranean Sea. The impact of the extracts on growth and adhesion of the target bacteria was studied. The selection of extracts was guided by the absence of toxicity and a significant anti-bioadhesion effect to better respect the regulatory evolution.

## 2. Results and Discussion

### 2.1. Bacterial Characterization

Bacteria from different origins (Atlantic Ocean, Indian Ocean and the Mediterranean Sea) were chosen as target organisms: bacteria are generally considered as the first and key colonising organisms in the biofouling formation. They were isolated from biofilms developed on dissimilar artificial surfaces immersed in seawater. The Neperian growth and generation rates as well as their morphologies and their ability to secrete exoproducts in Väätänen Nine-Salt Solution (VNSS) were studied at 20 °C (Table 1). The Neperian growth and generation rates were included between 0.36 and 0.87 h^−1^ and 0.52 and 1.47 h, respectively. The start of the stationnary phase was reached faster for Mediterranean and tropical strains as TC8 and MVV1 (5 h) than Atlantic strains that required more time (11 h).

**Figure 1:**
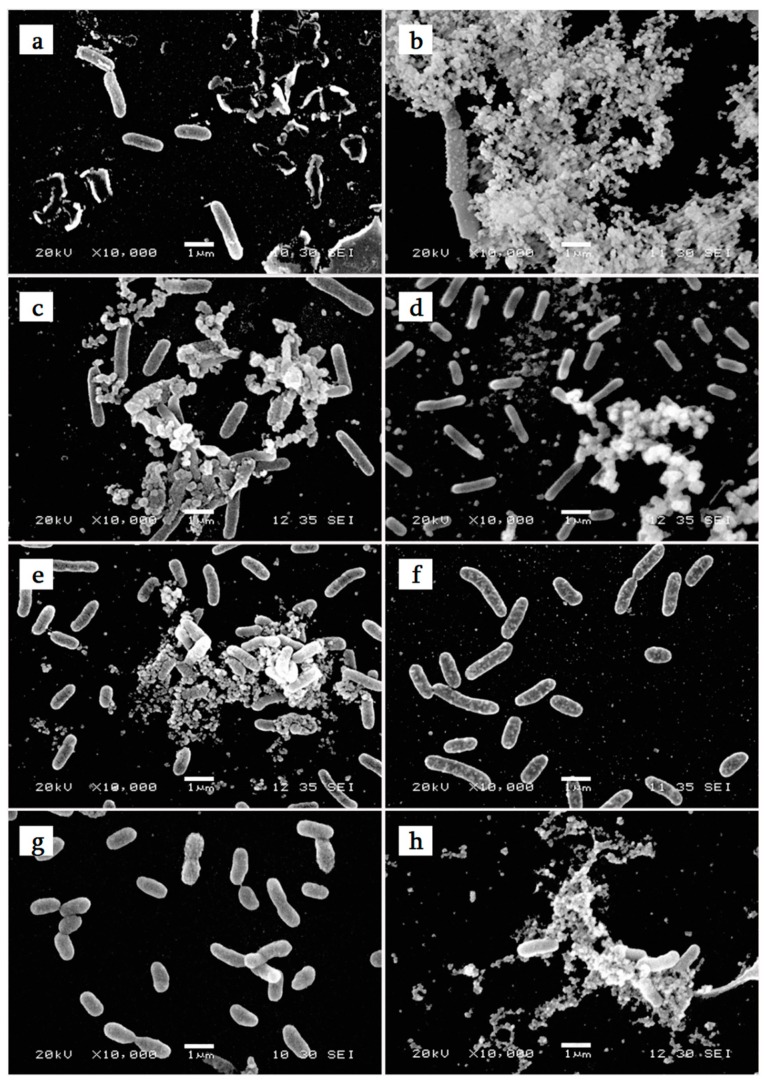
SEM photomicrographs of marine bacteria after 3 h adhesion on glass slides (ASW medium, 20 °C). (**a**) *Paracoccus* sp. 4M6, (**b**) *Bacillus* sp. 4J6, (**c**) *Pseudoalteromonas* sp. 5M6, (**d**) *Polibacter* sp. TC5, (**e**) *Pseudoalteromonas lipolytica* TC8, (**f**) *Shewanella* sp. TC11, (**g**) *Shewanella* sp. PVV6, (**h**) *Shewanella* sp. MVV1.

SEM studies made possible the description of the adhered bacterial morphology: cell size was measured (Figure 1, Table 1). The average length of marine bacteria was 1.23–2.05 µm. Bacteria of Atlantic Ocean origin showed a significantly longer size (Tukey’s HSD, *p* < 0.05) than tropical and Mediterranean strains. SEM pictures of marine bacteria showed the presence of extracellular polymeric substances (EPS) for some strains: 4J6 seem produce the highest amount, followed by 5M6, TC5, TC8 and MVV1 while 4M6, TC11 and PVV6 did not produce EPS. These EPS that coat bacterial cells, can modify their physicochemical characteristics such as surface charge and hydrophobicity. These changes should promote or not initial adhesion, cell aggregation and biofilm cohesion [30,31]. 

Hence, the capacity of adhesion of the eight strains might be different because, on the one hand, of their own physiological characteristics (appendices for example) and, on the other hand, because of the amounts and nature of the synthesized EPS.

### 2.2. Bacterial Adhesion

**Figure 2:**
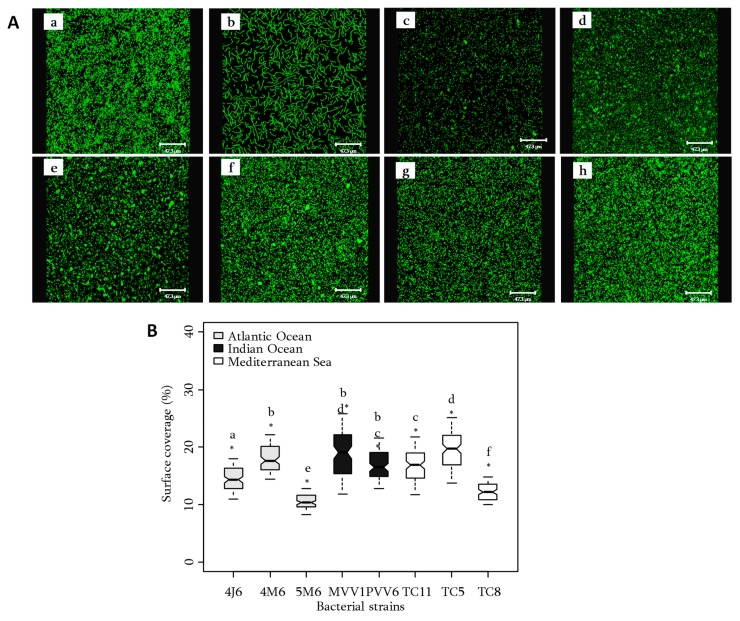
(**A**) CLSM observations of the marine bacterial adhesion with syto9® after 3 h (ASW medium, 20 °C). (**a**) *Paracoccus* sp. 4M6, (**b**) *Bacillus* sp. 4J6, (**c**) *Pseudoalteromonas* sp. 5M6, (**d**) *Polibacter* sp. TC5, (**e**) *Pseudoalteromonas lipolytica* TC8, (**f**) *Shewanella* sp. TC11, (**g**) *Shewanella* sp. PVV6, (**h**) *Shewanella* sp. MVV1. (**B**) Surface coverage (%) by marine bacteria. For each group significantly different from another, a letter (a, b, c, d, e, f or g) is assigned (Wilcoxon, * indicate *p* < 0.05).

The ability of the eight bacteria to adhere on glass slides was evaluated in a flow cell system [32]. A nutrient-free medium, such as artificial seawater (ASW), was selected to promote bacterial adhesion and disadvantage the cells multiplication. The observations were realised by confocal laser scanning microscopy (CLSM) after 3 h incubation at 20 °C and staining (Figure 2).

The spatial distribution of the bacterial cells on surface showed that all studied strains adhered on glass slides in a homogeneous way. However, some peculiarities could be advanced. For example, 4J6 showed chains of cells whereas aggregates were mainly observed for Mediterranean strains (TC11, TC5 and TC8) (Figure 2A). 

The quantification of adhesion is summarized in Figure 2B. Average surface coverages were included between 10 and 20%. Significant differences of adhesion (Wilcoxon, *p* < 0.05) were observed between strains. *Polibacter* sp. TC5 and *Shewanella* sp. MVV1 adhered significantly far more than other bacteria. On the contrary, *Pseudoalteromonas* sp. *5M6* and *Pseudoalteromonas lipolytical* TC8 showed the lowest surface coverages (10% average). No atypical value was observed under the conditions of the experiment. However, *Shewanella* sp. MVV1 showed a more heterogeneous adhesion as observed by a larger values distribution. This result could be explained by the formation of cell aggregates in the growth medium which decreased the repeatability of the bacterial inoculations. Results confirmed that marine bacterial strains exhibited various capacities to make biofilms [33]. However, no specificity between the adhesion ability, the EPS production and the geographical origin could be made.

### 2.3. Extracts of Tropical Microalgae

**Figure 3:**
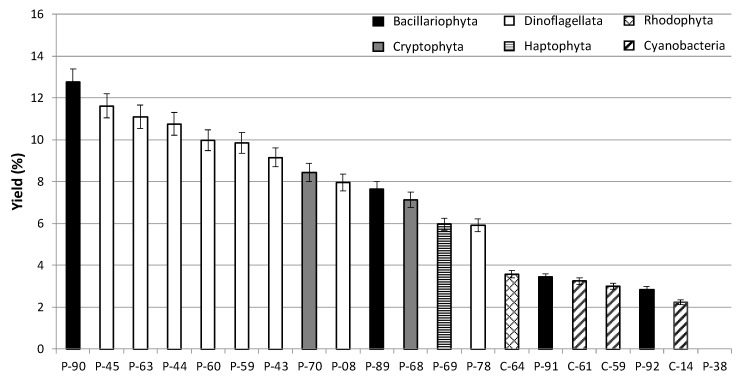
Extraction yield in methanol, in percent of dry matter, of tropical microalgae.

Methanolic extracts of twenty microalgae were prepared. Yields for each microalgal strain and each taxonomic group are given in Figure 3. Average yields were comprised between 2 and 13%. Dinoflagellates (Dinoflagellata) gave the higher extraction yields, whereas those of cyanobacteria were lower than 4%. The best yields were obtained for the diatom (Bacillariophyta) P-90 (12.8%), followed by the dinoflagellates P-45 (11.6%), P-63 (11.1%) and P-44 (10.7%).

### 2.4. Bioactivity of the Microalgal Extracts

The research of new strategies to inhibit adhesion and biofilm formation on surfaces immersed in seawater is based on the use of antiadhesive compounds without impact on the environment, so without bacteriostatic or bactericide effects. Hence, the efficient concentration inhibiting 50% of bacterial adhesion (EC_50_), the inhibitory concentration for 50% of the bacterial growth (IC_50_) and the lethal concentration for 50% of the bacteria (LC_50_) were determined.

#### 2.4.1. Impact on Bacterial Growth and Viability

Methanolic extract concentrations were 0.01, 0.1, 1, 10, 25, 50 and 100 μg mL^−1^. The determination of IC_50_ and LC_50_ was performed following the protocol described by Camps et al. [34]. The results were particularly interesting because none the microalgal extracts exhibited growth inhibition at 50 μg mL^−1^: IC_50_ values were higher than 50 μg mL^−1^ in all the case. Moreover, no toxicity was observed at the concentration of 50 μg mL^−1^ (LC_50_ > 50 μg mL^−1^).

IC_50_ values of booster biocides, such as diuron, irgarol and dichlofluanid are known to be particularly high against marine bacteria (>100 μg mL^−1^ generally) [35]. This requires the incorporation of large quantities of biocides in AF systems to obtain antibacterial efficiency. Indeed, bacteria are not the major targeted organisms of these compounds that are used to prevent the settlement of other colonizing organisms, such as algae, invertebrates or shellfishes. Other biocides, such as TBTO or SeaNine, showed lower IC_50_ values (<3 μg mL^−1^) [34]. To avoid any effect due to the toxicity of the microalgal extracts, a concentration of 50 μg mL^−1^ was adopted for the following experiments.

#### 2.4.2. Impact on Bacterial Adhesion

The anti-bioadhesion of the microalgal extracts were evaluated on the eight marine bacterial strains at a same concentration (50 μg mL^−1^) in a dynamic mode (flow cell). For each microalgal extract, the inhibition percentages of bacterial adhesion were determined by comparison between the standard conditions (without extract) and in the presence of the extract. All results were summarized in Table 2 In this table, microalgae were ranked *versus* their significant activity (*p* < 0.05, post-hoc HSD Tukey) from the best to the poorest activity. Four groups were identified. Microalgae belonging to group *a* have shown the highest bacterial inhibition. Three strains were in this group: P-78, P-43 and P-63 followed by P-60 (group b). The majority of extracts inhibited more than 50% bacterial adhesion of these strains. Microalgae classed in the group g showed the lowest activity: five strains were included in this group. The inhibition of bacterial adhesion was extremely low for most extracts. Other extracts had an intermediate activity (groups b–f).

**Figure 4:**
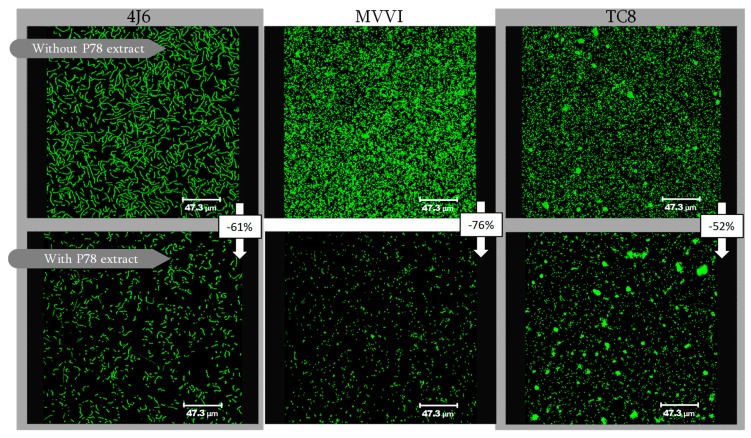
CLSM observations of three bacterial strains from Atlantic Ocean (*Bacillus* sp. 4J6), Indian Ocean (*Shewanella* sp. MVV1) and Mediterranean Sea (*Pseudoalteromonas lipolytica* TC8), with or without an extract from the P-78 strain (50 μg mL^−1^).

For illustration, the Figure 4 showed the results obtained for the more active extract (P-78) against one bacterial strain of each origin (Atlantic Ocean, Indian Ocean and Mediterranean See).

**Figure 5:**
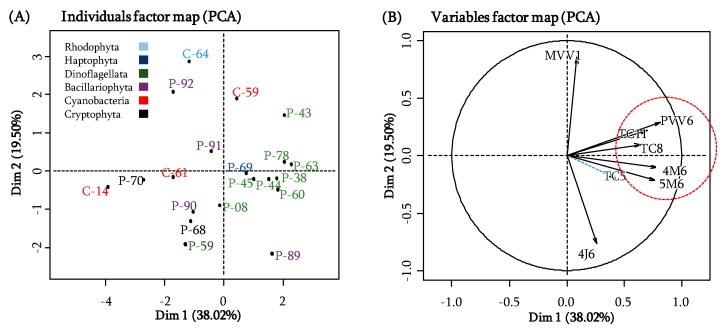
PCA analysis of bacterial adhesion. (**A**) the identification map of microalgae (ID), (**B**) the correlation circle with the bacterial strains that contributes the most to the formation of axis.

To observe possible clusterization of the different extracts depending on their activities on all the bacterial strains, a principal component analysis (PCA) was realized. It was based on the capacity of microalgal extracts to inhibit bacterial adhesion. The resulting plots showed that the two first components of this statistical model accounted for 57.52% (38.02% for the first component axis and 19.50% for the second one) of the total variance of the dataset (Figure 5). Thus, the first axis accounted for 38.02% and the second axis 19.50%. On the resulting score plot (Figure 5A), the first dimension allowed a gross discrimination between dinoflagellates extracts (excepted for P-59), positively correlated with the first axis, and the major part of the other extracts (excepted C-59, P-59, and P-89), negatively correlated with the first axis. Moreover, by comparison with Table 2, the most and the least active strains were situated positively and negatively on this first axis, respectively.

As noticed on the variable factor map, the first axis appeared to differentiate the anti-adhesion activity on most of the bacterial strains (*Shewanella* sp. PVV6, *Shewanella* sp. TC11, *Pseudoalteromonas* sp. TC8, *Paracoccus* sp. 4M6 and *Pseudoalteromonas* sp. 5M6), except *Shewanella* sp. MVV1 and *Bacillus* sp. 4J6 whose activities mostly explained the second axis. This indicates that except for the latter, most of the bacterial strains exhibited similar relative activity for the different microalgal extracts. Consequently, the first axis turned up to be a good proxy of the overall activity. Therefore, dinoflagellates were well clustered and separated from other less active strains.

Cyanobacteria and to a lesser extent diatoms are well known to be excellent sources of natural metabolites with antibacterial activity [36,37,38]. However, in this work, these taxonomic families were less efficient than Dinoflagellates. For example, the *Symbiodinium* sp. (P-78) extract inhibited the adhesion of *Bacillus* sp. 4J6 (Atlantic Ocean), *Shewanella* sp. MVV1 (Indian Ocean) and *Pseudoalteromonas lipolytica* TC8 (Mediterranean Ocean) at 61, 76 and 52% respectively as illustrated in Figure 4. This microalgal strain was the most active: the adhesion of seven bacterial strains out of the eight studied marine bacteria was inhibited (inhibition >50%) by its extract. Alone, the adhesion of TC11 was only inhibited at 40%. The three other strains (P-43, P-63 and P-60) corresponded to the genus *Amphidinium*. P-43 extract inhibited by more than 50% the adhesion of six of the eight bacterial strains assayed, whereas P-63 and P-60 extracts were highly active (inhibition >50%) against five bacterial strains. The genus *Amphidinium* and *Symbiodinium* belong to the taxonomic group of dinoflagellates. Dinoflagellates are known to be important sources of toxins such as macrolides, polyketides, polyols and polyether [39,40]. For example, zooxanthellamide Cs and symbioimine were identified from *Symbiodinium* sp. [41,42]. Amphidinin G, Karatungiols A and B, Carteraol E were isolated from *Amphidinium* sp. [43,44,45,46]. These compounds have been proven to show a wide range of biological activities (e.g., anti-resorptive, anti-inflammatory, antifungal, antiprotozoal and antibacterial).

Bacteria showed various sensitivity in the presence of microalgal extracts (Table 3). *Paracoccus* sp. 4J6, *Pseudoalteromonas* sp. 5M6, *Shewanella* sp. PVV6 and *Pseudoalteromonas lipolytica* TC8 were the more sensitive bacterial strains: their adhesion was inhibited by at least eleven microalgal extracts. Extracts acted indifferently against Gram-positive and Gram-negative bacteria, although Gram positive bacteria are known to show higher sensitivity to natural products than Gram negative bacteria [47]. Indeed, Gram negative bacteria show more resistance to natural compounds since the hydrophilic cell wall structure of these bacteria is constituted of a lipopolysaccharide that blocks the penetration of hydrophobic compounds and other extracts in the target cell membrane.

From the PCA described above, it was possible to observe a correlation between some bacterial strains and some microalgal extracts. In the loading plot (Figure 5B), a strong correlation was observed for *Shewanella* sp. PVV6, *Shewanella* sp. TC11, *Pseudoalteromonas lipolytica* TC8, *Paracoccus* sp. 4M6 and *Pseudoalteromonas* sp. 5M6 and most of the dinoflagellate extracts on the first axis whereas *Shewanella* sp. MVV1 and *Bacillus* sp. 4J6 allowed the discrimination of the other active extracts along the second axis (positively for C-59 and negatively for P-89). Noteworthy is the fact that anti-bioadhesion activities of most of the microalgal extracts against *Shewanella* sp. MVV1 and *Bacillus* sp. 4J6 were inversely correlated. Thus, three bacterial groups could be distinguished.

From these results, three bacterial strains and three microalgal extracts were selected for further investigations. *Shewanella* sp. MVV1, *Bacillus* sp. 4J6 and *Paracoccus* sp. 4M6 were representative of the three bacterial groups. As shown previously in Table 2, three extracts (P-43, P-60 and P-78) were the most active against the three selected bacteria. P-78 was active against the three strains whereas P-60 showed a remarkable inhibition of the adhesion of *Bacillus* sp. 4J6 (86%) and P-43 of *Shewanella* sp. MVV1 (82%). 

Then, the concentration needed to inhibit 50% of the bacterial adhesion was determined for each microalgal extract. These EC_50_ values (Table 4) ranged between 21 and 73 μg mL^−1^. These results were on the same order of magnitude as those obtained by Camps et al. [34] wherein EC_50_ values for commercial biocides against TC5, TC8 and 4M6 varied from 0.25 μg mL^−1^ to more than 160 μg mL^−1^. As described above, the three extracts have shown a high anti-adhesion activity against the three bacterial strains. However, their impacts were variable depending on the strain. *Bacillus* sp. 4J6 was significantly more sensitive to P-60 extract (*p* < 0.05, Wilcoxon), whereas *Paracoccus* sp. 4M6 and *Shewanella* sp. MVV1 were significantly more affected by P-78 extract (*p* < 0.05, Wilcoxon). *Paracoccus* sp. 4M6 seemed to be the most resistant strain.

These results confirmed that the anti-bioadhesion activity of these selected microalgal extracts relied on a mechanism rather than a toxic effect and underlined the potentiality of these natural ingredients as antifouling agents.

### 2.5. Which Microalgal Compounds Can Potentially Contribute to the Anti-adhesion Activity?

The global biochemical composition of the three selected microalgae has been determined in a previous work [48]. Firstly, the total lipid content was variable between strains. *Amphidinium* sp. were considered as high lipid producing strains: 42.62 and 24.70% of lipids from the dried biomass for P-60 and P-43 respectively, whereas *Symbidinium* strain P-78 produced only 2.83% of the lipids. Nevertheless, the distribution of lipids showed that polar lipids were present in majority in the microalgal extracts (90.65, 91.28 and 79.86% of the lipid content for P-78, P-60 and P-43 respectively). Free fatty acids (FAs) compositions were determined by gas chromatography. Results showed the dominant presence of docosahexaenoic acid (DHA, 22:6(n-3), 12.64, 20.84 and 9.47% of the total free FAs content for P-78, P-60, and P-43 respectively), eicosapentaenoic acid (EPA, 20:5(n-3), 20.40, 22.89 and 24.71% for P-78, P-60, and P-43 respectively) and palmitic acid (16:0, 30.70, 25,83 and 34,26% for P-78, P-60, and P-43, respectively) excepted for P-78 for which stearidonic acid (18:4(n-3), 19.80%) was also detected in high amounts. 

HR-MAS ^1^H-NMR analysis of microalgal extracts (spectra not shown) confirmed the presence of FAs and lipids (0–3 ppm), sugars (between 3 and 4.5 ppm) and the presence of peaks at 5.2–5.5 ppm, 2.8 ppm and 0.7–0.9 ppm characteristic of omega-3 FAs (unsaturated FAs) [49]. The high intensity of omega 3 FA characteristic ^1^H-NMR peaks indicated a large amount of these compounds in the three extracts. DHA and EPA are two major omega-3 polyunsaturated FAs which are found naturally at high levels in many marine organisms [50]. DHA and EPA have already demonstrated activities against pathogenic Gram-positive and Gram-negative bacteria and oral microorganisms [51,52,53,54,55]. Few studies are interested in the anti-biofilm activity of omega-3 FAs. Bacteriostatic and bactericidal actions of DHA and EPA against *Porphyromonas gingivalis* and *Fusobacterium nucleatum* has been previously shown [55]. However, the activity was strain-dependent. Moreover, an anti-biofilm activity (inhibition of biofilm formation and destruction of mature biofilm) with a decrease of cells viability was observed. Although the exact mechanism of action of FAs remains unknown, it affects various structures in microorganisms. Possibly, omega-3 FAs affect the integrity of the bacterial plasma membrane, thereby leading to cell damage and death [56,57]. Moreover, free FAs may affect the expression of bacterial virulence factors that are essential for the establishment of biofilms [55,56,57].

To confirm the bioactivity of EPA, the standard was tested against the marine bacteria *Bacillus* sp. 4J6, *Paracoccus* sp. 4M6 and *Shewanella* sp. MVV1. A decrease of bacterial adhesion of 82, 15 and 25% was obtained for *Bacillus* sp. 4J6, *Paracoccus* sp. 4M6 and *Shewanella* sp. MVV1, respectively, in the presence of 50 μg mL^−1^ of EPA.

Concerning palmitic acid, produced by the three microalgal strains, several studies have highlighted the antimicrobial potential of this compound [52,53,54]. Bazes et al. [58] found that palmitic acid (antibacterial activity at 44 μg mL^−1^) could be responsible for the antifouling activity observed in an active fraction isolated from the Phaeophyta *Sargassum muticum*.

Hence, the anti-bioadhesion activity of extracts could be attributed to the presence of FAs, and particularly of omega-3 FAs, in the microalgal extracts. Nevertheless, the low amount of lipids quantified from P-78 hypothesizes the presence of other compounds that could contribute to the observed activity. The chemical composition of these three microalgal extracts should be further investigated by combining several complementary approaches such as bioguided separation of active compounds and global annotation of their metabolome via LC-MS-based metabolomics and molecular networking.

## 3. Materials and Methods 

### 3.1. Bacterial Strains

The strains are listed in Table 5. *Paracoccus* (4M6), *Bacillus* (4J6) and *Pseudoalteromonas* (5M6) were isolated from the surface of glass covers immersed in Atlantic Ocean (Gulfe of Morbihan, France, 47°34′37″ N–2°44′54″ W) for 6 h at 1 m depth [59]. The bacterial strains (TC5, TC8, and T11) were isolated from the surface of silicone coupons immersed at 1 m depth in the Mediterranean Sea (Toulon Bay, France, 43°06′23″ N–5°57′17″ E) [33,34]. The tropical strains (MVV1 and PVV6) were isolated from the surface of glass covers immersed in the Indian Ocean at Sainte Marie and Le Port respectively (La Réunion) for 6 h. For this two latter stains, amplification of 16s rDNA was performed using the primers 63f and M1387R. PCR products (approximately 1500 bp) were cloned in TOPO TA Cloning Kit (Invitrogen, Carlsbad, CA, USA). Plasmid DNA were extracted with QIAprep Spin Miniprep Kit (Qiagen, Hilden, Germany) and sequenced by Genome Express (Cogenics, Meylan, France). Results were compared to bacterial genome data bank using tblastn with default parameters from NCBI website (http://blast.ncbi.nlm.nih.gov).

#### 3.1.1. Bacterial Growth and Morphology

The marine bacteria were grown on a rich medium: VNSS [60]. VNSS medium contains (g/L): peptone 1, yeast extract 0.5, glucose 0.5, amidon soluble 0.5, FeSO_4_·7H_2_O 0.01, Na_2_HPO_4_ 0.01, NaCl 17.6, Na_2_SO_4_ 1.47, NaHCO_3_ 0.08, KCl 0.25, KBr 0.04, MgCl_2_·6H_2_O 1.87, CaCl_2_·H_2_O 0.41, SrCl_2_·6H_2_O 0.01, H_3_BO_3_ 0.01. The cultures were inoculated at an optical density of 0.01 at 600 nm (OD_600_) and incubated at 20 °C whilst shaking (120 rpm). The Neperian growth rate (µ) was calculated as follows:

(1) $$N_{t}=N_{0}\cdot e^{µt}$$

where N_t_ and N_0_ are the number of bacteria at the t and initial times respectively. The generation time was determined as: G = Ln(2)/µ.

The bacterial morphology was observed by scanning electronic microscopy (SEM, 6460LV, JEOL Ltd., Tokyo, Japan) after adhesion on glass slide. Glass slides were introduced on Petri dish containing bacteria at 0.25 DO600 in ASW. After 3 h, slides were rinsed with ASW and immersed overnight in 3% glutaraldehyde solution. Then slides were dehydrated by several washings: phosphate buffer (10 min, 3 times); ethanol 70% (10 min, 3 times), ethanol 90% (10 min, 3 times), absolute ethanol (10 min, 3 times). Next, the samples were dried by the carbon dioxide critical point method (039 Critical Point Dryer, BAL-TEC, Balzers, Liechtenstein) and gold-coated prior to being observed by SEM.

#### 3.1.2. Bacterial Adhesion in Flow Cell

For adhesion tests, experiments were realized in a flow cell system [32]. The flow cell was prepared by sticking (Clear Super Silicone Sealant, 3M, St Paul, MN, USA) a microscope coverslip (24 × 50 mm, Knittel Glass, Braunschweig, Germany) slide which was the support of adhesion. After sterilization of the system by a flow of bleach (0.5%) during 24 h, a flow of minimum culture medium (ASW) was activated to clean and prepare the system for the bacterial injection.

The bacterial solution was prepared from a bacterial culture which was inoculated overnight. A dilution of the bacterial suspension was realized to inject bacteria at 0.25 OD_600_ ASW. Using a 1 mL syringe, 250 μL of the inoculum was injected in each channel. The flow cell was returned to facilitate the bacterial adhesion on the microscope coverslip. Bacteria were allowed to settle on the glass surface during 3 h in static conditions at 20 °C.

After incubation, the flow was activated during 30 min with the aim to remove free bacterial cells. Adhered bacteria were observed with Syto9^®^ nucleic acid stain (Invitrogen) at 5 μM (λ_excitation_ = 488 nm, λ_emission_ = 498–550 nm). The bacterial adhesion was observed with CLSM (Leica TCS-SP2, Heidelberg, Germany) by using a 40× oil immersion objective. The coverage surface was determined with a JAVA program. All the tested strains were replicated six times. Ten observations were realized on three canals. Thus, 180 data were obtained for each strains.

### 3.2. Microalgal Strains

Microalgae and cyanobacteria come from the PHYTOBANK collection at NEXA (La Reunion). The strains were collected in the Southwest Indian Ocean from 1992 to 2013 in the framework of various research programs in Réunion, Mayotte, Madagascar, Europa and Glorioso Islands (Table 6). 

Cyanobacteria were sampled using scuba-diving or snorkeling, supported by photographic documentation. At laboratory, samples were subdivided into three subsamples: one part of the sample was preserved with formaldehyde (4%) for later microscopic analysis; a second part was stored in ethanol for DNA analysis, and a third part was prepared for culture filing some filaments in agar plate and in liquid medium. Benthic microalgae were collected either directly from the different substrates or from marine organisms sampled by snorkeling or scuba-diving. 

#### 3.2.1. Isolation and Cultivation of Microalgae

Using an inverted microscope (Olympus IX71, Hamburg, Germany), microalgal cells were individually isolated by pipetting quickly after sampling, 3-fold rinsed in sterile seawater and transferred in plates with medium for growing. After some transfers to reach a high cell density, microalgal strains were transferred and cultured in glass tubes in F/2 medium (or F/2 medium supplemented with silica for diatoms strains) [61] at 26 °C with a 12 h:12 h photoperiod (around around 20–40 photon. m^−2^ s^−1^). The cultures were then maintained in the PHYTOBANK collection (Table 6).

#### 3.2.2. Isolation and Cultivation of Cyanobacteria

Raw biological samples were screened for cyanobacterial specimens using a microscopic lens and light microscope (Olympus BX51, Hamburg, Germany), and subsequently subjected to liquid culture enrichment, agar plates streaking or micromanipulation. Isolation and cultivation were performed using BG11 [61] or Z8 [62] media in sterile seawater and using 18 g/L agar concentration for agar plates. Seawater samples were filtered with glass fiber filters GF/F and autoclaved. The cultures were kept under a 12 h:12 h photoperiod (around 20–40 photon. m^−2^ s^−1^) at 26 °C. After several transfers of filaments, cyanobacteria strains were isolated and conserved at the PHYTOBANK collection (Table 6).

#### 3.2.3. Biomass Production 

For large-scale biomass production, isolates were grown in 250 mL to 5 L culture vessels with aeration under the same culture conditions than those previously described. After 3–4 weeks of growth, cells were harvested at stationary phase by filtration (biofilm forming species) or centrifugation for motile species (4000× *g* during 3 min; Heraeus^TM^ Megafuge^TM^ 1.0R centrifuge, Thermo Electron Co., Waltham, MA, USA), frozen at −20 °C and freeze-dried. For each strain, the total biomass was obtained from several cultures with successive harvesting, due to laboratory limitations for large-scale culture. 

#### 3.2.4. Extraction 

The microalgal extraction was dried and fine powdered. The resulting powder (0.5 g) was sequentially extracted three times by maceration into mixtures of dichloromethane/methanol (2:1, 1:1, and then 1:2 *v*/*v*; 3 × 25 mL) in an ultrasonic bath (15 min) at room temperature. The extracts were filtrated and concentrated under vacuum with 500 mg of RP-18 silica gel (Sepra C18-E, 50 μm Phenomenex, city, Le Pecq, France). The resulted residue was deposed on a solid phase extraction (SPE) cartridge (Phenomenex Strata C18-E, V = 6 mL, 500 mg silica) previously conditioned with methanol (10 mL) and water. Then, the solid crude was eluted with four washing: water (100%, 10 mL), a mixture of methanol/water (1:1, *v*/*v*, 10 mL), methanol (10 mL) and dichloromethane (10 mL). Four fractions were obtained and only the methanolic fraction was studied. Previous study has shown that methanolic fractions were the most active among the screening carried out [63]. The extraction yield was defined as the amount of methanolic fraction divided by the amount of dried algal biomass.

### 3.3. Extract Bioactivity

Methanolic fraction was evaporated to dryness. Then extract solutions from 1 to 50 µg/mL were prepared. The solvent used is water containing methanol (2%). In a preliminary study the effect of this solvent was evaluated on bacterial toxicity and adhesion. No impact was observed. 

#### 3.3.1. Anti-Adhesion Assay

Experiments were realized in flow cell [32] for the eight bacterial strains. Adhesion was performed on glass surface in three-channel flow cells (1 × 40 × 44 mm; Biocentrum, DTU, Amsterdam, Denmark). The system was assembled by sticking microscope coverslip slide (24 × 50 mm; Knittel Glass). After sterilization of the system by a flow of bleach (0.5%), then a flow of ASW (30 g/L) was activated to clean and prepare the system before bacterial injection. Channels were inoculated with 250 μL of overnight bacterial cultures diluted after centrifugation (4000× *g*, 10 min) in (a) ASW medium for the control, and (b) ASW medium containing microalgal extract (50 μg/mL). The flow cell was placed upside down to facilitate the cells adhesion on the glass. The incubation temperature was 20 °C and the incubation time was 3 h. After 3 h of adhesion, the flow at 120 μL/min was activated during 30 min to remove free bacterial cells. Cell adhesion was observed with CLSM. Adhered bacteria were observed with Syto 9 nucleic acid stain (5 μM). The overlap percentage was determined with a JAVA program (Université Bretagne Sud, Lorient, France). Experiments were realized in triplicate for each condition (27 observations in total). 

The percentage of inhibition was determined as following:

(2) $$\mathrm{Bacterial}\;\mathrm{inhibition}\;\left(\%\right)=\frac{\mathrm{Bacterial}\;\mathrm{adhesion}\;\mathrm{in}\;\mathrm{the}\;\mathrm{presence}\;\mathrm{of}\;\mathrm{algal}\;\mathrm{extract}}{\mathrm{Bacterial}\;\mathrm{adhesion}\;\mathrm{in}\;\mathrm{control}\;\mathrm{condition}}\times 100$$

#### 3.3.2. Determination of EC_50_ Values

The determination of EC_50_ values for the three selected microalgal strains was realized from bacterial adhesion observed by CLSM as described above. Methanolic extract concentrations were 0.01, 0.1, 1, 10, 25, 50 and 100 μg mL^−1^. After determination of a percentage of adhesion, a sigmoid dose-response curve was obtained and EC_50_ values were calculated (GraphPad Software, GraphPad Prism version 6.00 for Windows, La Jolla, CA, USA). Experiments were done in triplicate (27 observations in total). 

#### 3.3.3. Bacterial Growth Inhibition (EC_50_) and Viability Assays (LC_50_)

The determination of IC_50_ and LC_50_ values was performed following the protocol described by Camps et al. [34]. Briefly, 100 μL of algal extract were added in four wells of the microtiter plates (sterile transparent PS; Nunc, Fisher Scientific, Illkrich, France). Eight concentrations from 1 to 50 μg mL^−1^ were tested in triplicate. Then 100 μL of the bacterial suspension (OD_600_ = 0.1) was inoculated from an exponential bacterial culture on VNSS medium and the wells were filled out to 200 μL with VNSS. Turbidity (OD_600_) was measured every hour during 8 h. When the stationary phase was reached, resazurin (20 μM, Sigma-Aldrich, Saint Quentin Fallavier, France) was added on all the wells and fluorescence was measured after 2 h (λ_excitation_ = 535 nm, λ_emission_ = 595 nm) using the microplate fluorescence reader (TECAN, Magellan, Männedorf, Switzerland).

#### 3.3.4. Statistical Analysis

Statistical analyses of the parametric data were carried out using one or two-factor analysis variance (ANOVA). For no parametric tests, Kruskal Wallis and Wilcoxon tests were used. For all statistical analysis, the significant level was fixed to 95% (*p* < 0.05). To study the relations between the obtained data, PCA was performed and manipulated with the package FactoMineR package (factominer.free.fr/index_fr.html) under the R.3.1.2 environment. Classifications were performed with the function HCPC (K-means) of the FactoMineR package [48]. 

## Figures and Tables

**Table 1:** Characterization of bacterial growth and morphology.

		Growth				SEM Observation	
Geographical Origin	Strain	Taxonomy	Neperian Growth Rate (h^−1^)	Generation Rate (h)	Beginning of the Stationary Phase (h)	Length (μm)	EPS Production
Atlantic Ocean	4M6	*Paracoccus* sp.	0.72	0.97	11	1.76 ± 0.30	−
	4J6	*Bacillus* sp.	0.47	1.47	10	2.05 ± 0.29	++
	5M6	*Pseudoalteromonas* sp.	0.51	1.35	10	1.98 ± 0.26	+
Mediterranean Sea	TC5	*Polibacter* sp.	0.87	0.80	7	1.35 ± 0.16	+
	TC8	*Pseudoalteromonas lipolityca*	0.76	0.91	5	1.33 ± 0.20	+
	TC11	*Shewanella* sp.	0.72	0.96	8	1.63 ± 0.30	−
Indian Ocean	PVV6	*Shewanella* sp.	0.36	0.84	6	1.23 ± 0.07	−
	MVV1	*Shewanella* sp.	0.58	0.52	5	1.33 ± 0.11	+

−, no production; +, low production; ++, high production.

**Table 2:** Inhibition of bacterial adhesion (%) in the presence of tropical microalgal extracts (50 μg mL^−1^).

		Atlantic Ocean			Mediterranean Sea			Indian Ocean	
		*Bacillus* sp.	*Paracoccus* sp.	*Pseudoalt.*	*Polibacter* sp.	*Pseudoalt.*	*Shewanella* sp.	*Shewanella* sp.	*Shewanella* sp.
Strain	Group	4J6	4M6	5M6	TC5	TC8	TC11	PVV6	MVV1
P-78	a	61 ± 5	60 ± 3	87 ± 5	61 ± 4	52 ± 1	41 ± 4	61 ± 8	76 ± 4
P-43	a	41 ± 5	40 ± 10	71 ± 6	51 ± 10	62 ± 2	53 ± 7	91 ± 4	82 ± 10
P-63	a	38 ± 7	74 ± 1	68 ± 7	51 ± 3	78 ± 4	46 ± 1	70 ± 5	36 ± 10
P-60	b	86 ± 3	46 ± 4	63 ± 6	37 ± 7	66 ± 6	49 ± 3	69 ± 6	56 ± 3
P-89	c	68 ± 5	44 ± 7	64 ± 9	70 ± 4	32 ± 3	45 ± 5	86 ± 6	−22 ± 2
P-45	d	67 ± 9	25 ± 3	49 ± 6	49 ± 3	51 ± 1	42 ± 5	70 ± 7	48 ± 2
P-69	d	54 ± 2	59 ± 9	86 ± 5	17 ± 15	43 ± 23	16 ± 15	53 ± 27	52 ± 8
C-59	e	23 ± 2	19 ± 1	60 ± 8	12 ± 4	69 ± 3	40 ± 3	63 ± 8	72 ± 2
P-38	e	58 ± 1	60 ± 2	72 ± 4	12 ± 12	67 ± 2	53 ± 2	75 ± 2	29 ± 2
P-44	e	41 ± 5	48 ± 1	64 ± 26	41 ± 9	78 ± 5	48 ± 7	57 ± 5	19 ± 5
P-90	f	75 ± 9	−16 ± 7	29 ± 5	28 ± 8	34 ± 6	49 ± 12	17 ± 6	22 ± 7
P-91	f	60 ± 2	46 ± 7	−20 ± 5	1 ± 9	33 ± 11	32 ± 9	72 ± 13	48 ± 5
P-08	f	48 ± 4	20 ± 4	43 ± 9	28 ± 3	56 ± 4	44 ± 4	25 ± 4	1 ± 5
P-59	f	57 ± 2	11 ± 4	51 ± 10	45 ± 4	57 ± 7	−50 ± 7	34 ± 4	−1 ± 5
C-64	f	−7 ± 6	2 ± 6	28 ± 2	26 ± 5	22 ± 6	20 ± 5	64 ± 7	84 ± 7
P-68	g	52 ± 4	46 ± 3	65 ± 7	19 ± 16	19 ± 12	3 ± 12	−6 ± 13	19 ± 9
P-70	g	36 ± 3	−7 ± 11	−41 ± 11	49 ± 3	42 ± 2	−3 ± 9	−6 ± 10	26 ± 7
C-61	g	51 ± 5	−16 ± 5	−6 ± 4	47 ± 5	73 ± 8	21 ± 8	−18 ± 2	43 ± 3
C-14	g	55 ± 7	5 ± 3	−2 ± 5	−11 ± 8	−37 ± 5	−6 ± 15	−12 ± 3	43 ± 4
P-92	g	7 ± 2	2 ± 2	−41 ± 4	37 ± 4	49 ± 2	21 ± 6	41 ± 3	62 ± 1

Letters correspond to extracts groups differentiated significantly (*p* < 0.05, HSD Tukey) from highly (a) to lowly (g) active.

**Table 3:** Extract number inhibiting 50% bacterial adhesion.

Strain	4M6	4J6	5M6	TC5	TC8	TC11	PVV6	MVV1
Extracts Number	4	12	11	4	11	2	12	6

**Table 4:** Effect of selected microalgal extracts on the adhesion of a selection marine bacteria. R^2^ corresponded to the fitting quality for the dose response curve (>0.8).

Microalgal Strain		Bacterial Strain		
		4J6	4M6	MVV1
P-43	EC_50_ (μg mL^−1^)	32 ± 6	73 ± 2	31 ± 7
	Hill slope	Median	Median	Low
	R^2^	0.9 ± 0.2	0.9 ± 0.1	0.9 ± 0.3
P-60	EC_50_ (μg mL^−1^)	25 ± 1	57 ± 13	39 ± 10
	Hill slope	High	Median	Low
	R^2^	0.9 ± 0.1	0.9 ± 0.1	0.9 ± 0
P-78	EC_50_ (μg mL^−1^)	37 ± 4.	35 ± 6	21 ± 1
	Hill slope	Median	Median	Low
	R^2^	0.9 ± 0.1	0.8 ± 0.2	0.9 ± 0

For the Hill slopes, “high” corresponded to a value lower than −5, “low” a value higher than −2 and median an intermediate value.

**Table 5:** Bacterial strains used in the study.

Strain	Taxonomy	Origin	Gram	Source
4M6	*Paracoccus* sp.	Atlantic Ocean	−	[59]
4J6	*Bacillus* sp.		+	
5M6	*Pseudoalteromonas* sp.		−	
TC5	*Polibacter* sp.	Mediterranean Sea	−	
TC8	*Pseudoalteromonas lipolityca*		−	[33,34]
TC11	*Shewanella* sp.		−	
PVV6	*Shewanella* sp.	Indian Ocean	−	This study
MVV1	*Shewanella* sp.		−	

**Table 6:** Description of the microalgal strains used in this study.

Strain Code ^a^	Phylum	Family	Genus	Species	Origin	Growth Medium
C-64	Rhodophyta	ND ^b^	Porphyridium	sp.	Glorioso	BG11 ^c^
P-69	Haptophyta	Pavlovaceae	*Pavlova*	sp.	Glorioso	F/2 ^d^
P-38	Dinoflagellata	Gymnodiniaceae	*Amphidinium*	sp.	Reunion	F/2
P-43			*Amphidinium*	sp.	Europa	F/2
P-44			*Amphidinium*	*massartii*	Glorioso	F/2
P-45			*Amphidinium*	sp.	Glorioso	F/2
P-59			*Amphidinium*	sp.	Madagascar	F/2
P-60			*Amphidinium*	*operculatum*	Madagascar	F/2
P-63			*Amphidinium*	sp.	Madagascar	F/2
P-08		Prorocentraceae	*Prorocentrum*	*lima*	Reunion	F/2
P-78		Symbiodiniaceae	*Symbiodinium*	sp. (Clade D)	Reunion	F/2
P-91	Bacillariophyta	Bacillariaceae	*Navicula*	mollis	Reunion	F/2 + Si ^e^
P-92			*Navicula*	sp.	Reunion	F/2 + Si
P-89			*Psammodictyon*	cf. *constrictum*	Reunion	F/2 + Si
P-90			*Nitzschia*	sp.	Reunion	F/2 + Si
C-59	Cyanobacteria	ND	ND ^b^	sp5	Glorioso	BG11
C-61			ND ^b^	sp6	Glorioso	BG11
C-14			ND ^b^ (LPP-group)	sp. LPP1	Mayotte	BG11
P-70	Cryptophyta	ND	ND^b^	sp1	Glorioso	F/2
P-68			ND^b^	sp3	Glorioso	F/2

^a^ PHYTOBANK code. ^b^ not determined, ^c^ Blue Green Medium, ^d^ Gillard’s medium, ^e^ silica.
